# Supplementary figures and images for: Mining of Thousands of Prokaryotic Genomes Reveals High Abundance of Prophages with a Strictly Narrow Host Range
Source: mSystems. 2022 Jul 26;7(4):e00326-22. doi: 10.1128/msystems.00326-22 (PMC9426530; doi:10.1128/msystems.00326-22)

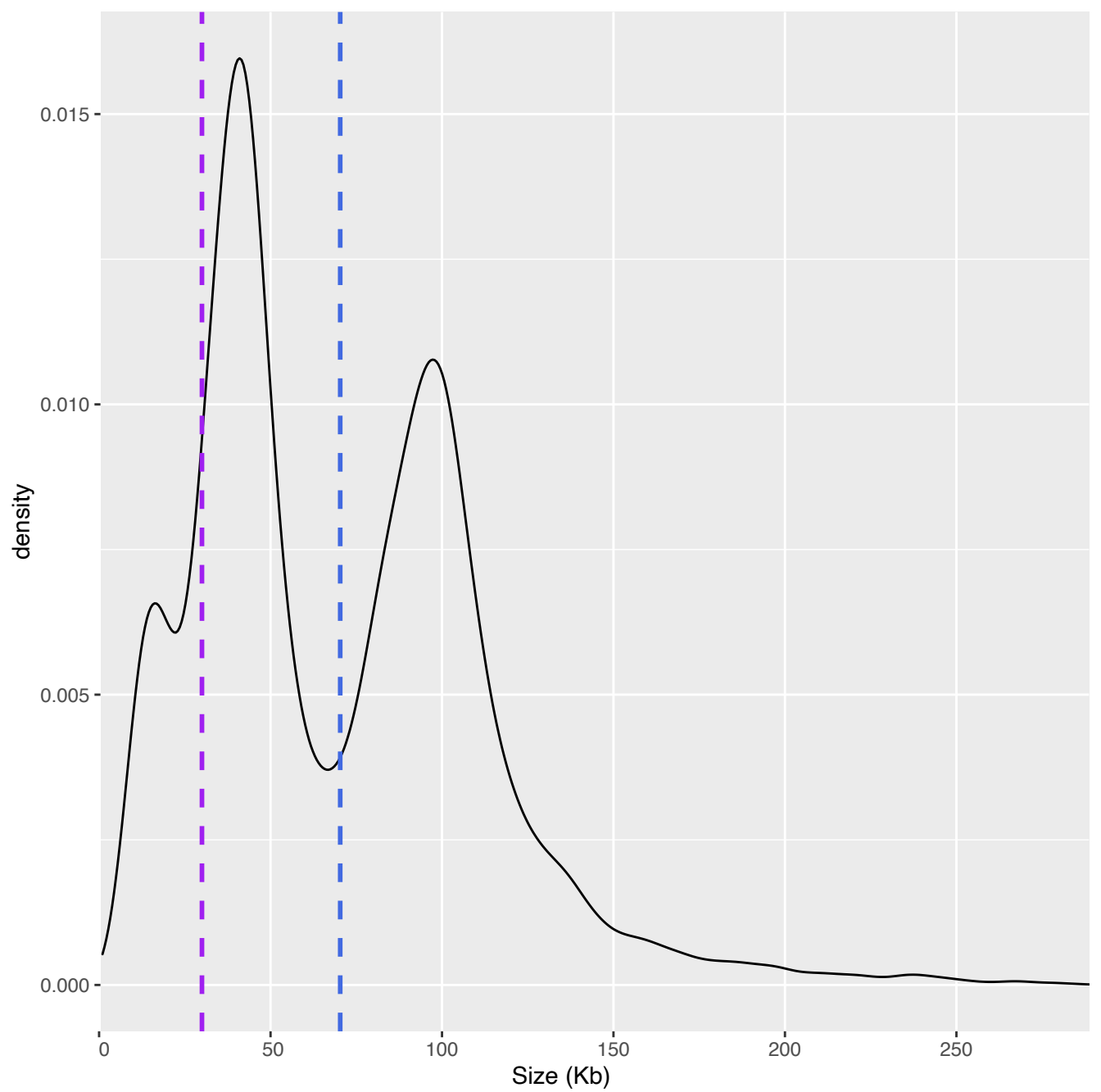

Supplement: FIGURE S1 [file msystems.00326-22-s0002.pdf]

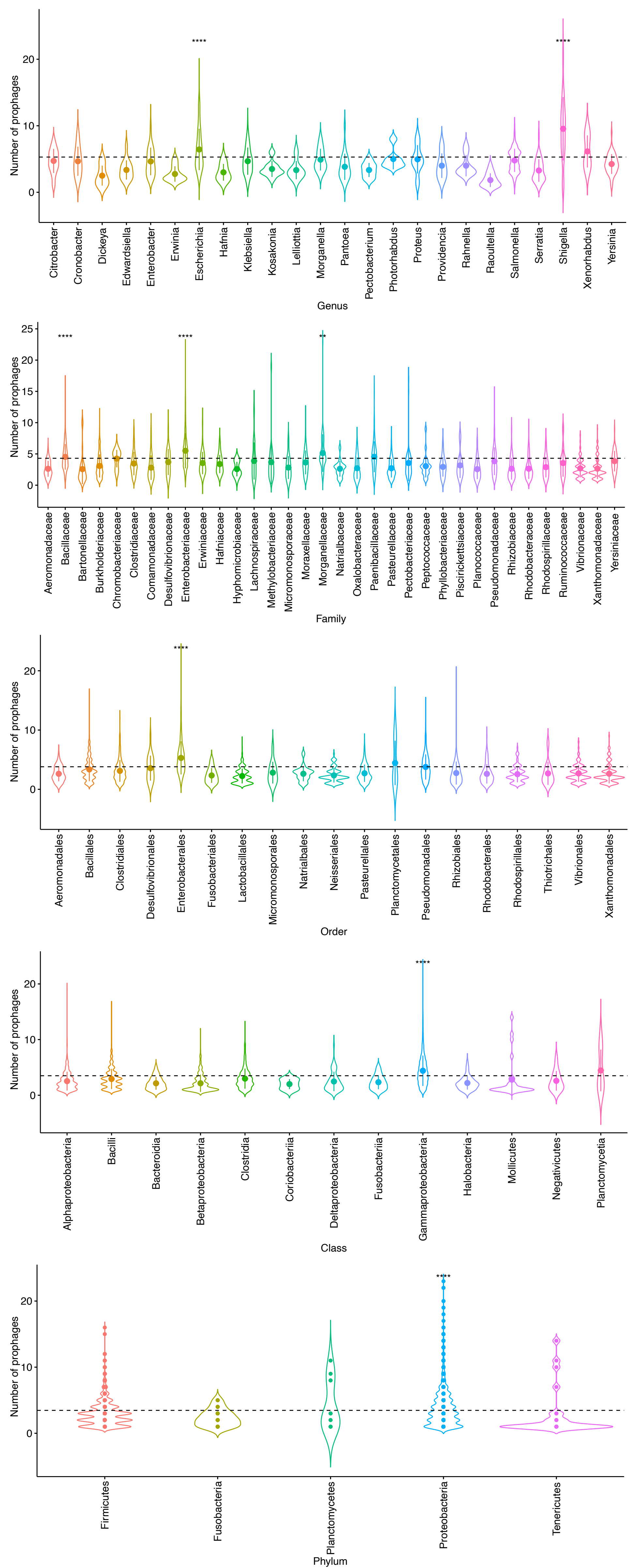

Supplement: FIGURE S2 [file msystems.00326-22-s0003.pdf]

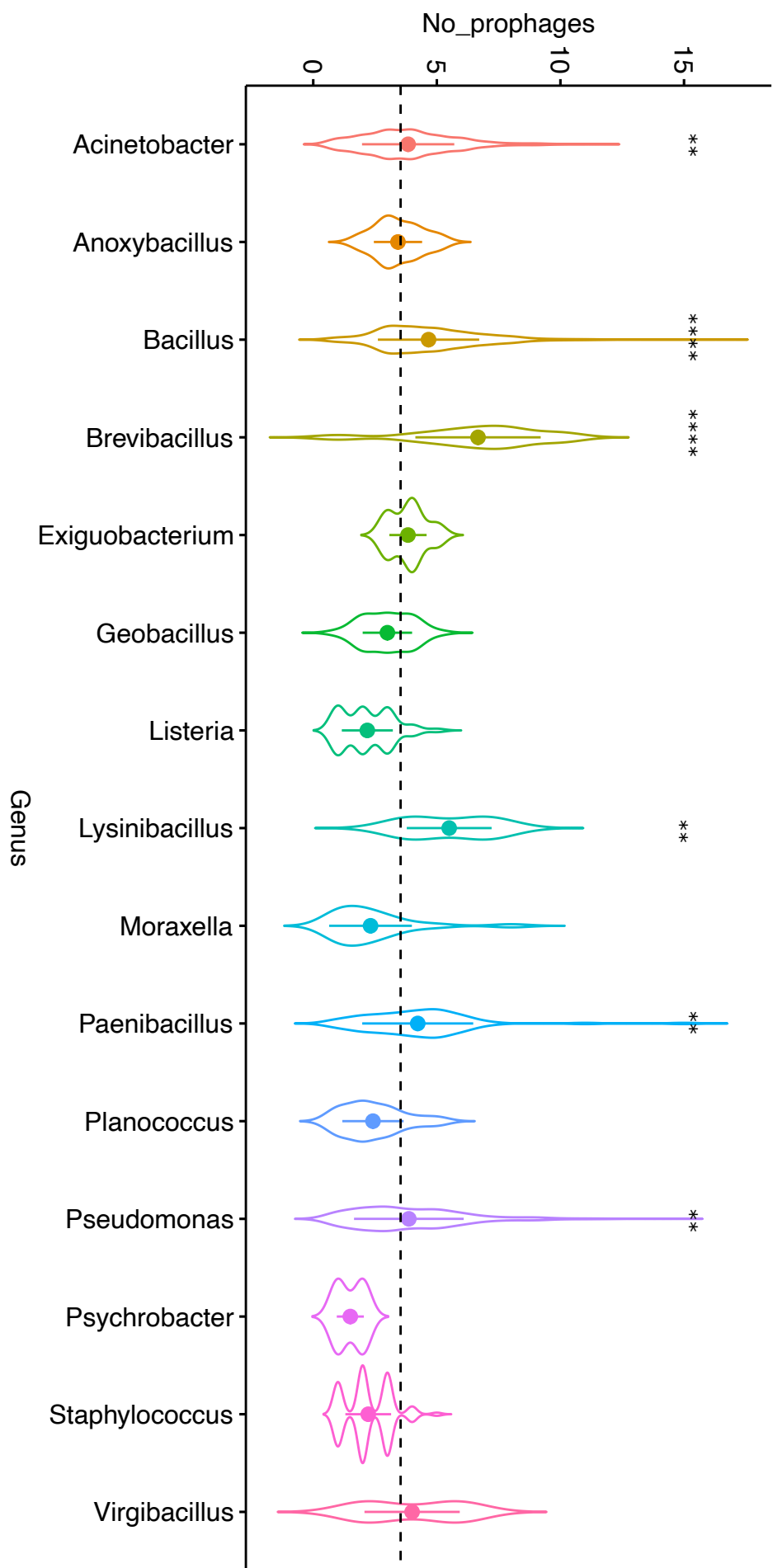

Supplement: FIGURE S3 [file msystems.00326-22-s0004.pdf]

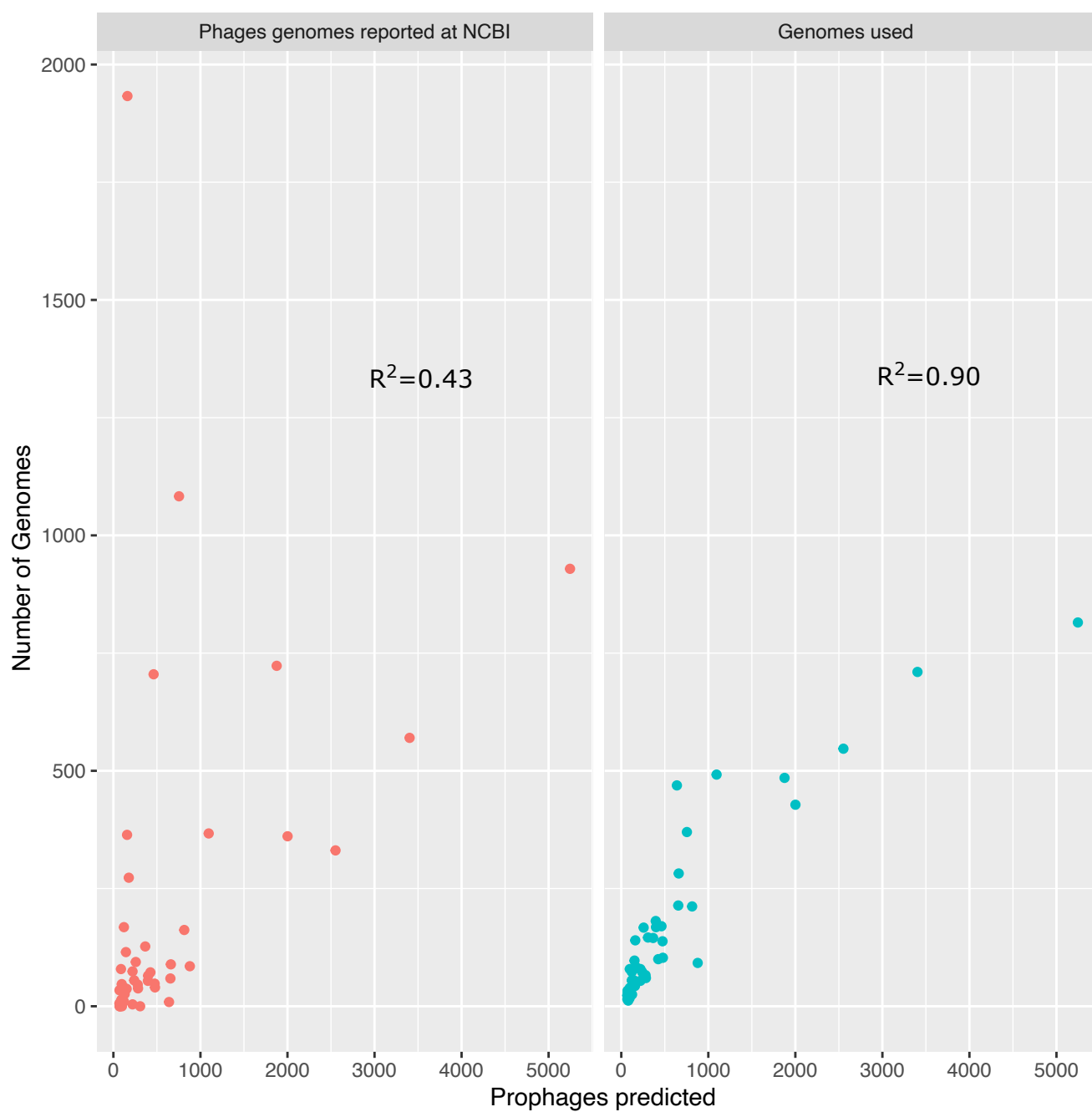

Supplement: FIGURE S4 [file msystems.00326-22-s0005.pdf]

VC\_94

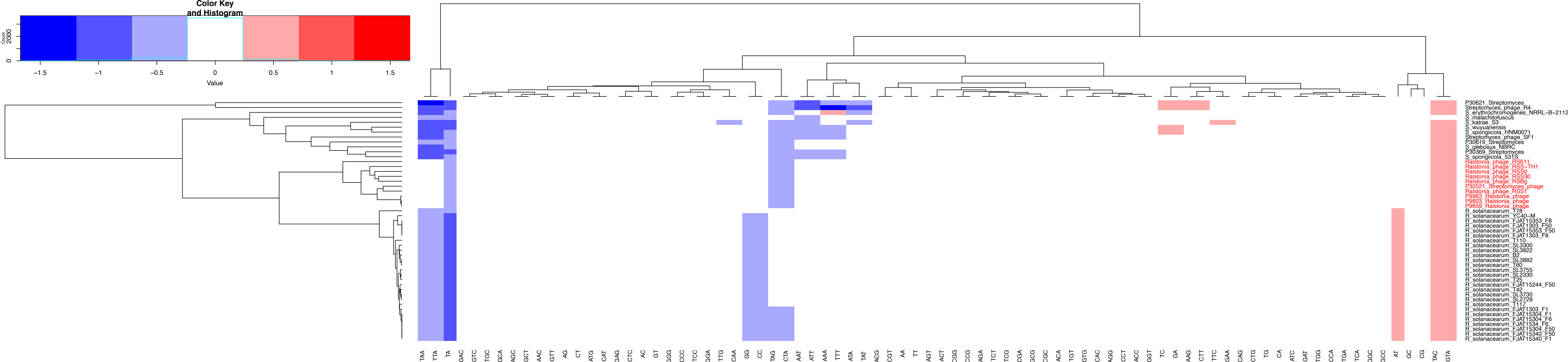

VC\_1254

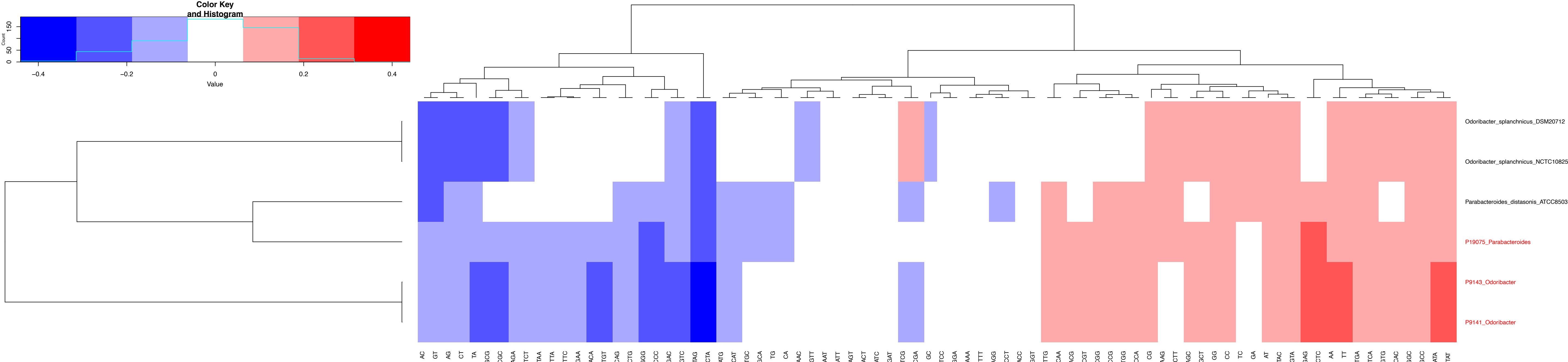

Supplement: FIGURE S6 [file msystems.00326-22-s0009.pdf]

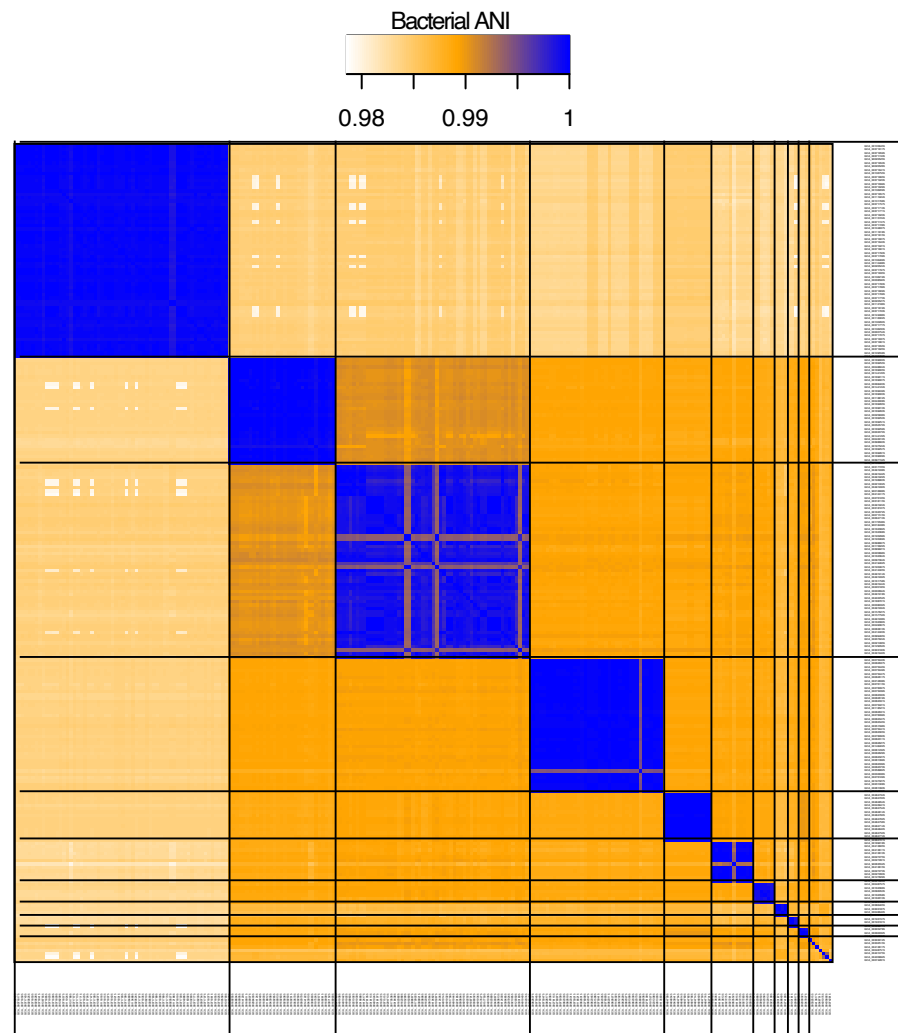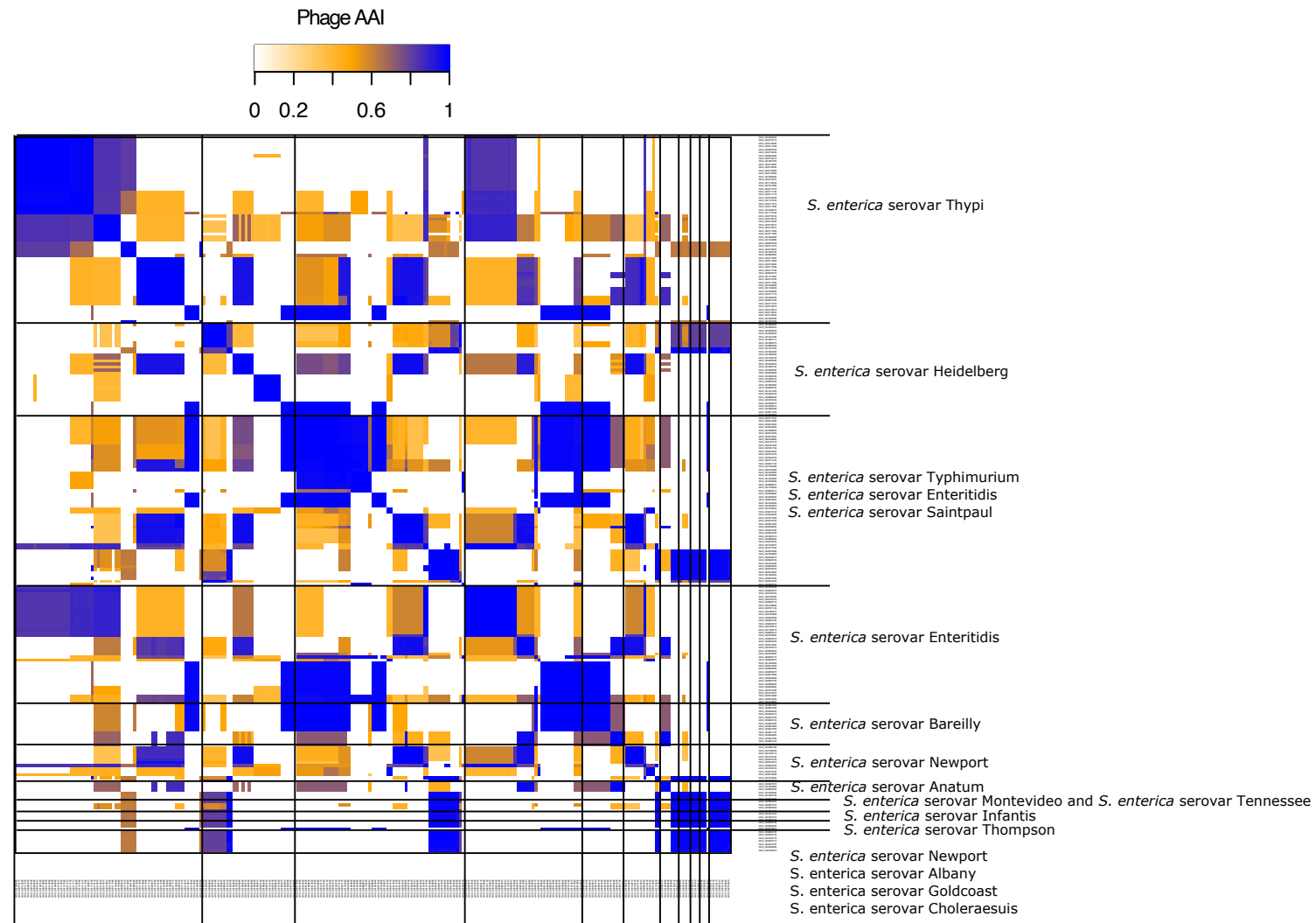

Supplement: FIGURE S7 [file msystems.00326-22-s0010.pdf]
