# Supplementary material for: Mining of Thousands of Prokaryotic Genomes Reveals High Abundance of Prophages with a Strictly Narrow Host Range
Source: mSystems. 2022 Jul 26;7(4):e00326-22. doi: 10.1128/msystems.00326-22 (PMC9426530; doi:10.1128/msystems.00326-22)
Supplement: FIGURE S5 [file msystems.00326-22-s0008.pdf]

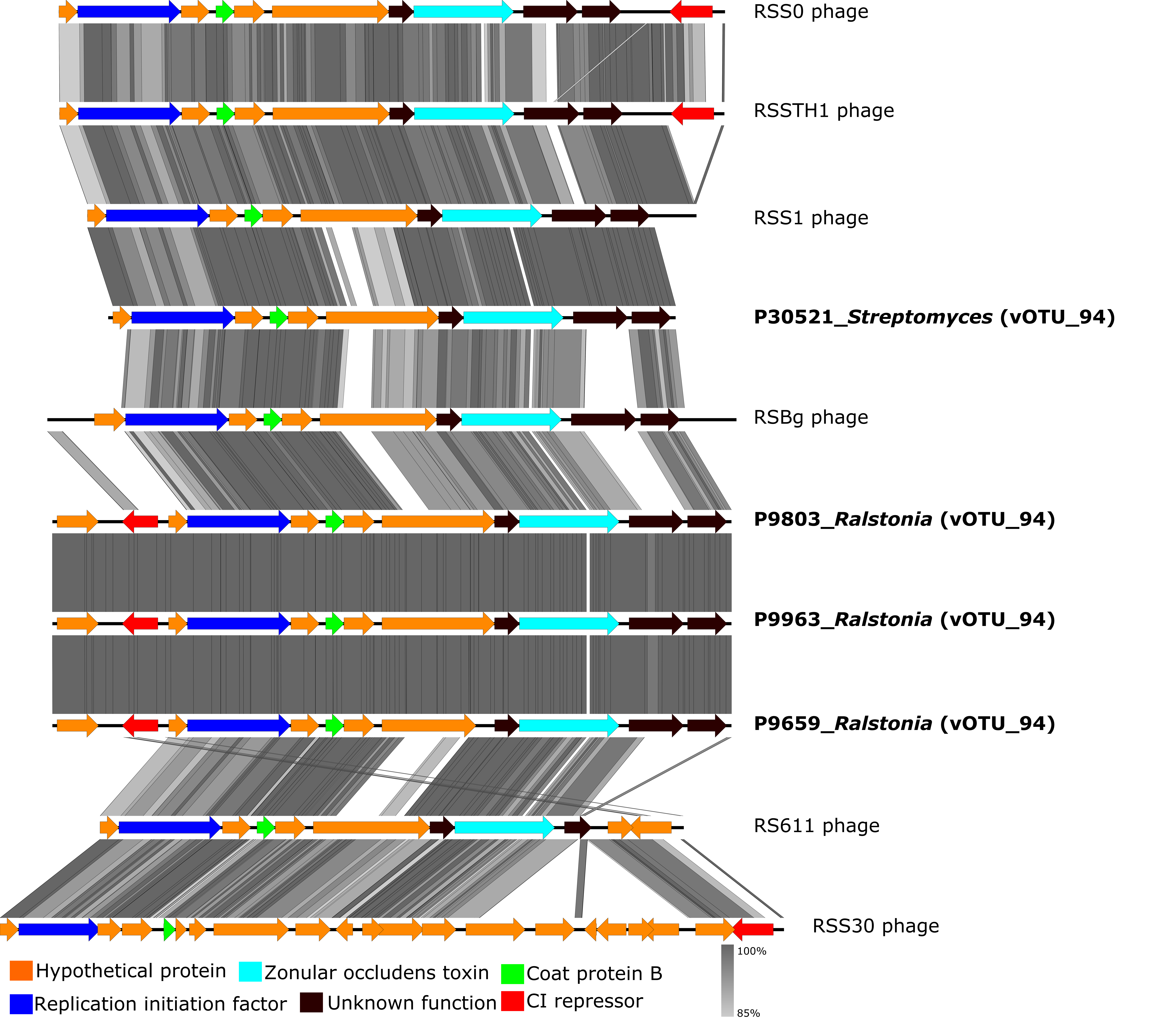
RSS0 phage
RSSTH1 phage
RSS1 phage
P30521_*Streptomyces* (vOTU_94)
RSBg phage
P9803_*Ralstonia* (vOTU_94)
P9963_*Ralstonia* (vOTU_94)
P9659_*Ralstonia* (vOTU_94)
RS611 phage
RSS30 phage
Hypothetical protein
Zonular occludens toxin
Coat protein B
Replication initiation factor
Unknown function
CI repressor
100%
85%
