# Supplementary material for: Mining of Thousands of Prokaryotic Genomes Reveals High Abundance of Prophages with a Strictly Narrow Host Range
Source: mSystems. 2022 Jul 26;7(4):e00326-22. doi: 10.1128/msystems.00326-22 (PMC9426530; doi:10.1128/msystems.00326-22)
Supplement: Supplementary file 4 [file msystems.00326-22-s0007.docx]

	Pathogens			Unassigned				
Genus	Number of Genomes	Number of Prophages	SD prophage abundance	Number of Genomes	Number of Prophages	SD prophage abundance	*p*- value	*p*- value adjsuted
Enterobacter	49	268	1.95	44	175	1.89	6.28E-05	0.008597965
Acinetobacter	54	234	1.46	36	113	1.89	0.00028052	0.038431718
Pseudomonas	22	114	2.36	58	190	1.97	0.00028874	0.039557739
